# Molecular epidemiology of *Staphylococcus aureus* in African children from rural and urban communities with atopic dermatitis

**DOI:** 10.1186/s12879-021-06044-4

**Published:** 2021-04-13

**Authors:** Gillian O. N. Ndhlovu, Regina E. Abotsi, Adebayo O. Shittu, Shima M. Abdulgader, Dorota Jamrozy, Christopher L. Dupont, Avumile Mankahla, Mark P. Nicol, Carol Hlela, Michael E. Levin, Nonhlanhla Lunjani, Felix S. Dube

**Affiliations:** 1 Department of Molecular and Cell Biology, Faculty of Science, University of Cape Town, Cape Town, South Africa; 2 Institute of Infectious Disease & Molecular Medicine, University of Cape Town, Cape Town, South Africa; 3 Department of Pharmaceutical Microbiology, School of Pharmacy, University of Health and Allied Sciences, Ho, Ghana; 4 Department of Microbiology, Obafemi Awolowo University, Ile-Ife, Osun State Nigeria; 5 Institute of Medical Microbiology, University Hospital Münster, Münster, West Germany; 6 Department of Pathology, Division of Medical Microbiology, Faculty of Medicine and Health Sciences, Stellenbosch University, Tygerberg, South Africa; 7 Parasites and Microbes Programme, Wellcome Sanger Institute, Hinxton, UK; 8 J. Craig Venter Institute, La Jolla, California USA; 9 Department of Medicine and Pharmacology, Division of Dermatology, Walter Sisulu University, Umtata, South Africa; 10 Division of Infection and Immunity, School of Biomedical Sciences, University of Western Australia, Perth, Australia; 11 Department of Paediatrics, Division of Paediatric Allergy, University of Cape Town, Cape Town, South Africa

## Abstract

**Background:**

*Staphylococcus aureus* has been associated with the exacerbation and severity of atopic dermatitis (AD). Studies have not investigated the colonisation dynamics of *S. aureus* lineages in African toddlers with AD. We determined the prevalence and population structure of *S. aureus* in toddlers with and without AD from rural and urban South African settings.

**Methods:**

We conducted a study of AD-affected and non-atopic AmaXhosa toddlers from rural Umtata and urban Cape Town, South Africa. *S. aureus* was screened from skin and nasal specimens using established microbiological methods and clonal lineages were determined by *spa* typing. Logistic regression analyses were employed to assess risk factors associated with *S. aureus* colonisation.

**Results:**

*S. aureus* colonisation was higher in cases compared to controls independent of geographic location (54% vs. 13%, *p* < 0.001 and 70% vs. 35%, *p* = 0.005 in Umtata [rural] and Cape Town [urban], respectively). Severe AD was associated with higher colonisation compared with moderate AD (86% vs. 52%, *p* = 0.015) among urban cases. Having AD was associated with colonisation in both rural (odds ratio [OR] 7.54, 95% CI 2.92–19.47) and urban (OR 4.2, 95% CI 1.57–11.2) toddlers. In rural toddlers, living in an electrified house that uses gas (OR 4.08, 95% CI 1.59–10.44) or utilises kerosene and paraffin (OR 2.88, 95% CI 1.22–6.77) for heating and cooking were associated with increased *S. aureus* colonisation. However, exposure to farm animals (OR 0.3, 95% CI 0.11–0.83) as well as living in a house that uses wood and coal (OR 0.14, 95% CI 0.04–0.49) or outdoor fire (OR 0.31, 95% CI 0.13–0.73) were protective. *Spa* types t174 and t1476, and t272 and t1476 were dominant among urban and rural cases, respectively, but no main *spa* type was observed among controls, independent of geographic location. In urban cases, *spa* type t002 and t442 isolates were only identified in severe AD, t174 was more frequent in moderate AD, and t1476 in severe AD.

**Conclusion:**

The strain genotype of *S. aureus* differed by AD phenotypes and rural-urban settings. Continued surveillance of colonising *S. aureus* lineages is key in understanding alterations in skin microbial composition associated with AD pathogenesis and exacerbation.

**Supplementary Information:**

The online version contains supplementary material available at 10.1186/s12879-021-06044-4.

## Introduction

Atopic dermatitis (AD) is a common childhood inflammatory skin disease that frequently presents in early childhood [1]. The prevalence of AD is high in developed countries where it affects 10–20% of children [2]. However, recent epidemiological data indicate an increase in the prevalence of AD among children in developing countries, including South Africa [3–5]. The increasing prevalence of AD and allergy is also associated with urbanisation with a lower prevalence and microbial-related protective environmental factors noted in rural areas [3, 6]. Patients with AD usually suffer from persistent or relapsing itchy and dry eczematous skin lesions with inflammation and increased susceptibility to cutaneous *Staphylococcus aureus* (*S. aureus*) colonisation associated with perturbation of the skin microbial community [7, 8]. In addition to skin colonisation, *S. aureus* has also been reported to colonise the nasal cavity as a primary reservoir for extra-nasal auto-transmission [9]. Skin and nasal *S. aureus* colonisation have been demonstrated in both AD patients and healthy individuals; however, a higher colonisation density and prevalence have been described in AD patients [9]. *S. aureus* colonisation has also been associated with AD pathogenesis [10], with colonisation preceding the clinical onset of AD in early childhood [11]. *S. aureus* produces a variety of virulence factors, including superantigens, proteases, as well as dermolytic and cytolytic toxins which contribute to the progression of AD [12]. Nonetheless, other staphylococcal species, including *S. epidermidis* and *S. haemolyticus* have been implicated in the pathophysiology of AD by the degradation of epidermal structural proteins [13, 14]. Molecular epidemiological studies have shown that while colonisation occurs in both AD patients and healthy individuals, the genetic background of colonising *S. aureus* strains differ across AD disease phenotypes and may influence disease pathogenesis and severity [1, 15]. We hypothesised that geographic location affects *S. aureus* colonisation in AD and health through distinct environmental exposures. Here, we report the prevalence and genotypes of *S. aureus* from skin and nasal samples of AmaXhosa AD and non-AD toddlers in rural and urban South African settings. In addition, we evaluated the risk factors for *S. aureus* colonisation in each geographic location.

## Materials and methods

### Study design, setting and population

#### Participant recruitment

**Fig. 1:**
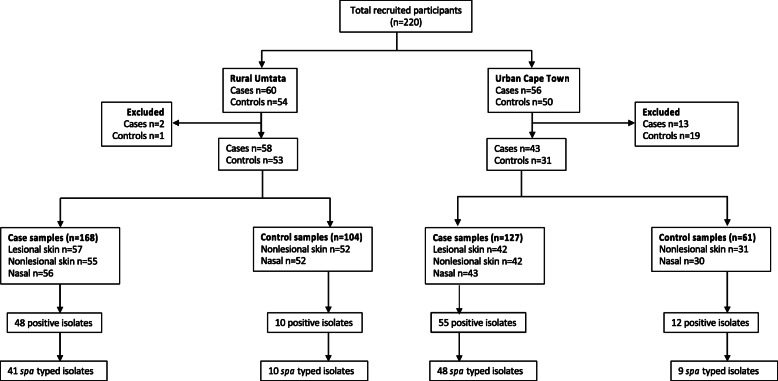
Flow chart of participants’ sample processing. Eleven participants (eight cases and three controls) had one unavailable specimen for either the lesional skin, nonlesional skin or anterior nares

We conducted a cross-sectional study of 220 toddlers with and without AD aged 12–36 months (overall mean age 22.4 months; standard deviation 0.54 months) from February 2015 to May 2016 (Fig. 1) [6, 16]. Urban control subjects (*n* = 50) were recruited as a sub-study from non-allergic, non-food-sensitised subjects participating in the South African Food Allergy (SAFFA) study at randomly selected creches in the Cape Town metropole. As creches are rarely found in the rural district, rural controls (*n* = 54) were recruited from toddlers of eligible age from the areas surrounding 10 district community health clinics in the rural Mqanduli district of Umtata. Patients with moderate to severe AD (*n* = 56) were recruited from the Department of Paediatric Dermatology of the Red Cross War Memorial Children’s Hospital in Cape Town and rural cases (*n* = 60) from the Department of Dermatology, Nelson Mandela Academic Hospital, in Umtata. AD was clinically diagnosed by a dermatologist using the validated UK Working Party diagnosis criteria for AD [17]. Disease severity was determined using the objective SCORAD (SCORing of Atopic Dermatitis) index into moderate (15–40) and severe (> 40) [18]. Guardians completed a questionnaire aimed at determining environmental exposures as previously described [19].

#### Specimen collection and processing

Sterile Copan nylon-tipped flocked swabs (Cat. no. 516C; Copan Italia, Brescia, Italy) were used to collect samples from lesional (i.e., most active area of eczematous skin with acute and/or chronic changes) and non-lesional skin (i.e., area with the most normal-appearing skin – usually the back). The swab was pre-moistened with sterile distilled water and a 4 cm^2^ area of the skin lesion was swabbed for at least 1 min in a non-overlapping manner. In addition, nasal swabs were collected from all participants to determine the *S. aureus* carriage status according to previously described methodology [20]. The collected swabs were immediately placed into 1 ml skim milk-tryptone-glucose-glycerol (STGG), transported at 4 °C to the laboratory within two hours of collection and frozen at − 80 °C for subsequent batch processing. All lesional, non-lesional, and nasal swabs stored in STGG were allowed to thaw at room temperature, vortexed for 30 s and 100 μl was inoculated onto Mannitol Salt Agar (MSA) (National Health Laboratory Services [NHLS], Green Point Media Laboratory Cape Town, South Africa), and aerobically incubated at 37 °C for 48 h. Isolates that were positive for both mannitol fermentation and DNase production were presumptively identified as *S. aureus* [21].

#### Nucleic acid extraction

Recovered *S. aureus* isolates were aerobically subcultured onto 2% sheep blood agar at 37 °C overnight. Genomic DNA extraction was completed using a modified heat lysis method [22]. Briefly, colonies were re-suspended in AVE buffer (Qiagen, Hilden, Germany) instead of phosphate-buffered saline and centrifuged at 13,000 g for two minutes. The supernatant containing genomic DNA was diluted in AVE buffer depending on the initial DNA concentration to a final concentration range of 20-70 ng/μl.

#### Molecular identification of the *S. aureus* isolates

Isolates presumptively identified as *S. aureus* were screened for the thermonuclease (*nuc*) gene using species-specific primers as previously described [23].

#### Molecular characterisation

*S. aureus* isolates were characterised by staphylococcus protein A (*spa*) typing targeting the variable X-region of the gene using the conventional primers *spa*-1113F/*spa*-1514R [24, 25]. Isolates that failed to yield a *spa* amplicon or had poor sequence quality were re-analysed using alternative *spa* primers T3F/1517R or 1095F/1517R [26, 27]. Clustering was based on their genetic relatedness to *spa*-clonal complexes (*spa*-CCs) using the Based Upon Repeat Pattern (BURP) clustering algorithm of the Ridom Staph Type software (Ridom GmbH, Münster, Germany) [28]. PCR detection of the *nuc* gene was performed to rule out misidentification of isolates that failed to yield a *spa* amplicon [23].

### Statistical analysis

All data analyses were performed using Stata version SE16.0 (1985–2019 StataCorp LP, Texas, USA). The significance threshold for all analyses was 0.05. Univariate and multivariate analyses to assess risk factors for *S. aureus* colonisation were performed using logistic regression and presented as odd ratios (OR) and adjusted ORs (aOR) reported with a 95% confidence interval (CI). The level of statistical significance in the logistic regression analysis was determined by a Chi-square test. Variables that were significant determinants for colonisation were included in the multivariate logistic regression model. Comparison of categorical data was performed by Chi-squared test unless stated otherwise. Comparison of means was performed using the t-test for two independent samples reported with a standard deviation (SD). Participants with missing data were excluded from the analyses relating to that variable.

## Results

### *S. aureus* colonisation in cases and controls

A total of 185 (84 controls and 101 cases) toddlers were assessed for *S. aureus* colonisation (Table 1). Thirty-five participants were excluded (missing specimen) from the study analysis (Fig. 1). Of these, 79 (43%) were colonised with *S. aureus* in at least one of the sampled body sites. There was an overall higher prevalence of colonisation among urban participants compared to rural participants (55% [41/74] vs. 34% [38/111], *p* = 0.006). *S. aureus* was commonly detected from cases compared to controls in both rural (54% [31/58] vs. 13% [7/53], *p* < 0.001 and urban settings (70% [30/43] vs. 35% [11/31], *p* = 0.005). Furthermore, cases were more frequently colonised on non-lesional skin compared to controls, and this was independent on geographic location (Additional file 1: Table S1). Among cases, colonisation was more common on lesional skin compared to non-lesional skin (rural: *p* = 0.035 and urban: *p* = 0.021) and the anterior nares (rural: *p* = 0.008). The prevalence of colonisation was common among urban cases with severe disease (86% [19/22] vs. 52% [11/21], *p* = 0.015), however, this was not associated with the site of colonisation (Additional file 2: Table S2). Overall, these findings show that geographic location influences the dynamics of *S. aureus* colonisation on skin and nares in AD and non-AD, and this is dependent on the site of colonisation and disease severity.

**Table 1:** Participant characteristics of atopic dermatitis cases and healthy controls

Explanatory variable	Umtata				Cape Town			
	Total, ***n*** (%)	Case, ***n*** (%)	Control, ***n*** (%)	***p***-value	Total, ***n*** (%)	Case, ***n*** (%)	Control, ***n*** (%)	***p-***value
Total	111 (100)	58 (52)	53 (48)	0.502^a^	74 (100)	43 (58)	31 (42)	0.049^a^
Age (months)								
Mean [standard deviation]	21.27 [7.15]	21.03 [7.41]	21.53 [6.90]	0.718	24.19 [7.37]	23.98 [7.44]	24.48 [7.38]	0.773
Sex								
Female	42 (39)	24 (43)	18 (34)	0.431	36 (49)	19 (44)	17 (55)	0.480
Male	67 (61)	32 (57)	35 (66)		38 (51)	24 (56)	14 (45)	
AD severity								
Moderate	23 (40)	23 (40)	–		21 (49)	21 (49)	–	
Severe	35 (60)	35 (60)	–		22 (51)	22 (51)	–	
Atopic disease								
Food allergy	11 (10)	10 (17)	1 (2)	**0.009**	9 (12)	9 (21)	0 (0)	**0.008**
Asthma	0 (0)	0 (0)	0 (0)		1 (1)	1 (3)	0 (0)	1.000
Allergic rhinitis	7 (8)	1 (2)	6 (11)	0.242	1 (1)	1 (3)	0 (0)	1.000
Mode of birth								
Caesarean section	25 (23)	14 (24)	11 (21)	0.821	33 (46)	20 (49)	13 (42)	0.637
Vaginal	86 (77)	44 (76)	42 (79)		39 (54)	21 (51)	18 (58)	
Breastfeeding	35 (32)	10 (17)	25 (47)	**0.001**	9 (12)	7 (16)	2 (6)	0.288
Antibiotic exposure	92 (82)	49 (83)	43 (81)	0.810	54 (72)	30 (70)	24 (77)	0.598
Immunisation status								
Complete	107 (96)	56 (95)	52 (98)	0.620	64 (86)	33 (77)	31 (100)	0.004
Incomplete	4 (4)	3 (5)	1 (2)		10 (14)	10 (23)	0 (0)	
Large family ^a^	62 (55)	30 (52)	32 (60)	0.445	24 (32)	14 (33)	10 (32)	1.000
Animal exposure	93 (84)	39 (67)	53 (100)	**0.001**	2 (3)	2 (6)	0 (0)	0.495
Parental education								
Primary	8 (7)	2 (3)	6 (11)	**0.001**	1 (1)	1 (2)	0 (0)	**0.025**
Secondary	70 (63)	31 (53)	39 (74)		33 (45)	14 (33)	19 (61)	
Tertiary	31 (28)	25 (43)	6 (11)		40 (54)	28 (65)	12 (39)	
Other	2 (2)	0 (0)	2 (4)		0 (0)	0 (0)	0 (0)	
Maternal factors								
Animal exposure	96 (86)	44 (76)	52 (98)	**0.001**	4 (60)	4 (11)	0 (0)	0.120
Pregnant smoking	1 (1)	0 (0)	1 (2)	0.482	3 (45)	0 (0)	3 (10)	0.094
Smoking	1 (1)	0 (0)	1 (2)	0.477	4 (6)	1 (3)	3 (10)	0.324
Asthma	2 (2)	2 (3)	0 (0)	0.496	6 (8)	4 (10)	2 (6)	1.000
Allergic rhinitis	4 (4)	4 (7)	0 (0)	0.120	5 (68)	4 (10)	1 (3)	0.387
Atopic dermatitis	2 (2)	2 (3)	0 (0)	0.496	3 (4)	2 (5)	1 (3)	1.000
Food allergy	3 (3)	2 (3)	1 (2)	1.000	1 (1)	1 (2)	0 (0)	1.000
Paternal factors								
Smoking	15 (14)	9 (16)	6 (12)	0.589	20 (31)	11 (31)	9 (31)	1.000
Asthma	3 (3)	3 (5)	0 (0)	0.245	0 (0)	0 (0)	0 (0)	
Allergic rhinitis	3 (3)	3 (5)	0 (0)	0.245	7 (10)	7 (17)	0 (0)	0.018
Atopic dermatitis	1 (1)	1 (2)	0 (0)	1.000	2 (3)	2 (5)	0 (0)	0.505
Food allergy	1 (1)	1 (2)	0 (0)	1.000	1 (1)	1 (2)	0 (0)	1.000
Household factors								
Electricity + gas	69 (62)	56 (97)	13 (25)	**0.001**	66 (99)	35 (97)	31 (100)	1.000
Kerosene + paraffin	64 (58)	44 (76)	20 (38)	**0.001**	43 (64)	21 (58)	22 (71)	0.317
Paraffin	38 (34)	6 (10)	32 (60)	**0.001**	0 (0)	0 (0)	0 (0)	
Indoor fire	4 (4)	2 (3)	2 (4)	1.000	0 (0)	0 (0)	0 (0)	
Outdoor fire	49 (44)	12 (21)	37 (70)	**0.001**	0 (0)	0 (0)	0 (0)	
Wood + fire	31 (28)	4 (7)	27 (51)	**0.001**	0 (0)	0 (0)	0 (0)	

Bold text indicates statistical significance. *AD* atopic dermatitis, *CI* confidence interval, *IQR* interquartile range; ^a^ Large family is arbitrarily defined as 7 or more members living within one household

### Risk factors associated with *S. aureus* colonisation across the locations

The effect of various risk factors on colonisation with *S. aureus* in toddlers from both locations using logistic regression are shown in Tables 2 and 3, for rural and urban toddlers, respectively. The univariate analysis models showed that having AD was associated with colonisation in both rural (OR 7.54, 95% CI 22.92–19.47) and urban (OR 4.2, 95% CI 1.57–11.2) toddlers. Also, living in an electrified house that utilises gas (OR 4.08, 95% CI 1.59–10.44) and kerosene and paraffin (OR 2.88, 95% CI 1.22–6.77) for heating and cooking were associated with an increased risk of *S. aureus* among the rural toddlers. Surprisingly, exposure to farm animals (OR 0.3, 95% CI 0.11–0.83) as well as living in a house that uses wood and coal (OR 0.14, 95% CI 0.04–0.49) and outdoor fire (OR 0.31, 95% CI 0.13–0.73) were associated with lower odds of colonisation. In the multivariate model of rural toddlers, having AD (aOR 8.02, 95% CI 1.28–50.37) was retained as a risk factor for *S. aureus* colonisation, while living in a house that uses wood and coal for cooking and heating (aOR 0.02, 95% CI 0.02–0.99) remained protective against *S. aureus* colonisation. No regression analysis was performed for urban toddlers because only AD showed an association with *S. aureus*. In summary, the findings highlight the importance of the immediate environment, or exposome, in *S. aureus* colonisation.

**Table 2:** Unconditional logistic regression analysis of child, parental, domestic and environmental characteristics associated with *S. aureus* colonisation in Umtata participants

Explanatory variable	Colonised ^a^, *n* (%)	Not colonised, *n* (%)	OR [95% CI]	*p-*value	aOR [95% CI]	*p*-value
AD: case	31 (28)	27 (24)	7.54 [2.92–19.47]	**0.000**	8.02 [1.28–50.37]	**0.026**
Sex: male	21 (19)	46 (42)	0.74 [0.33–1.67]	0.469	0.83 [0.32–2.16]	0.696
Child characteristics						
Breastfeeding	10 (9)	25 (23)	0.69 [0.29–1.63]	0.395	1.46 [0.48–4.47]	0.503
Allergic rhinitis	1 (1)	6 (7)	0.43 [0.05–3.79]	0.449	Excluded	
Asthma ^§^	0 (0)	0 (0)	Omitted ^d^		Excluded	
Food allergy	5 (5)	6 (5)	1.69 [0.48–5.95]	0.413	Excluded	
Mode of delivery: vaginal	29 (26)	57 (51)	0.9 [0.36–2.29]	0.833	Excluded	
Incomplete immunisation status	2 (2)	2 (2)	1.97 [0.27–14.58]	0.506	Excluded	
Antibiotic exposure	33 (30)	58 (52)	1.71 [0.57–5.12]	0.34	1.54 [0.39–6]	0.536
Large family size ^b^	15 (14)	35 (32)	0.71 [0.32–1.57]	0.395	0.94 [0.36–2.44]	0.903
Animal exposure ^c^	27 (24)	65 (59)	0.3 [0.11–0.83]	**0.021**	0.53 [0.11–2.54]	0.429
Fossil fuel exposure						
Electricity + gas	31 (28)	38 (34)	4.08 [1.59–10.44]	**0.003**	0.35 [0.05–2.47]	0.295
Kerosene + paraffin	28 (25)	36 (32)	2.88 [1.22–6.77]	**0.015**	0.69 [0.19–2.49]	0.571
Indoor fire	1 (1)	3 (3)	0.63 [0.06–6.27]	0.694	Excluded	
Outdoor fire	10 (9)	39 (35)	0.31 [0.13–0.73]	**0.008**	0.54 [0.17–1.67]	0.283
Wood + coal	3 (3)	28 (25)	0.14 [0.04–0.49]	**0.002**	0.14 [0.02–0.99]	**0.048**
Maternal factors						
Allergic rhinitis	0 (0)	4 (4)	Omitted ^d^		Excluded	
Asthma	1 (1)	1 (1)	1.95 [0.12–32]	0.641	Excluded	
Atopic dermatitis	1 (1)	1 (1)	1.95 [0.12–32]	0.641	Excluded	
Food allergy	1 (1)	2 (2)	0.96 [0.08–10.93]	0.973	Excluded	
Smoking	0 (0)	1 (1)	Omitted		Excluded	
Pregnant smoker	0 (0)	1 (1)	Omitted		Excluded	
Animal exposure ^c^	31 (28)	65 (59)	0.55 [0.18–1.64]	0.28	1.93 [0.37–10.16]	0.438
Paternal factors						
Allergic rhinitis ^§^	0 (0)	3 (3)	Omitted		Excluded	
Asthma ^§^	1 (1)	2 (2)	0.96 [0.08–10.93]	0.973	Excluded	
Atopic dermatitis ^§^	0 (0)	1 (1)	Omitted		Excluded	
Food allergy ^§^	0 (0)	1 (1)	Omitted		Excluded	
Smoking	5 (5)	10 (9)	0.94 [0.3–2.98]	0.267	Excluded	

*AD* atopic dermatitis, *OR* odds ratio, *aOR* adjusted odds ratio, *CI* confidence interval; ^§^ No within group variance; ^a^ Colonisation with *Staphylococcus aureus*; ^b^ Large family size is arbitrarily defined as 7 or more members within a household; ^c^ Animal exposure refers to farm animals; ^d^ Independent variables omitted due to dependency in the regression model

**Table 3:** Unconditional logistic regression analysis of child, parental, domestic and environmental characteristics associated with *S. aureus* colonisation in Cape Town participants

Explanatory variable	Colonised ^a^, *n* (%)	Not colonised, *n* (%)	OR [95% CI]	*p-*value
AD: case	30 (41)	13 (18)	4.2 [1.57–11.2]	**0.004**
Sex: male	19 (26)	19 (25)	0.74 [0.33–1.67]	0.469
Child characteristics				
Breastfeeding	6 (8)	3 (4)	1.71 [0.39–7.45]	0.472
Atopic dermatitis				
Allergic rhinitis	1 (1)	0 (0)	Omitted ^d^	
Asthma ^§^	1 (1)	0 (0)	Omitted ^d^	
Food allergy	6 (8)	3 (4)	1.71 [0.39–7.45]	0.472
Mode of delivery: vaginal	22 (31)	17 (24)	0.95 [0.37–2.43]	0.921
Incomplete immunisation status	8 (11)	2 (3)	3.76 [0.74–19.09]	0.11
Antibiotic exposure	31 (42)	23 (31)	1.35 [0.48–3.77]	0.57
Large family size ^b^	9 (12)	8 (11)	0.88 [0.3–2.61]	0.816
Animal exposure ^c^	1 (1)	1 (1)	0.86 [0.05–14.3]	0.915
Fossil fuel exposure				
Electricity + gas	36 (54)	30 (45)	Omitted ^d^	
Kerosene + paraffin	20 (30)	23 (34)	0.43 [0.15–1.23]	0.116
Indoor fire	0 (0)	0 (0)	Omitted ^d^	
Outdoor fire	0 (0)	0 (0)	Omitted ^d^	
Wood + coal	0 (0)	0 (0)	Omitted ^d^	
Maternal factors				
Allergic rhinitis	0 (0)	5 (7)	Omitted ^d^	
Asthma	3 (4)	3 (4)	0.76 [0.14–4.06]	0.751
Atopic dermatitis	1 (1)	2 (3)	0.38 [0.03–4.33]	0.432
Food allergy	1/73	0 (0)	Omitted ^d^	
Smoking	3 (4)	1 (1)	2.65 [0.26–26.82]	0.41
Pregnant smoker	2 (3)	1 (1)	1.76 [0.15–20.45]	0.65
Animal exposure ^c^	3 (5)	1 (2)	2.64 [0.26–26.76]	0.412
Paternal factors				
Allergic rhinitis ^§^	5 (7)	2 (3)	2.08 [0.38–11.52]	0.4
Asthma ^§^	0 (0)	0 (0)	Omitted ^d^	
Atopic dermatitis ^§^	2 (3)	0 (0)	Omitted ^d^	
Food allergy ^§^	1 (1)	0 (0)	Omitted ^d^	
Smoking	13 (20)	7 (11)	1.86 [0.62–5.54]	0.267

*AD* atopic dermatitis, *OR* odds ratio, *CI* confidence interval; ^§^ No within group variance; ^a^ Colonisation with *Staphylococcus aureus*. ^b^ Large family size is arbitrarily defined by more than 6 members within a household; ^c^ Animal exposure refers to exposure to farm animals; ^d^ Independent variables omitted due to dependency in the regression model

### Clonal lineages of recovered *S. aureus* isolates

A total of 125 skin and nasal *S. aureus* isolates were recovered from cases and controls, however, only 108 isolates were characterised by *spa* typing (Fig. 1). Seventeen isolates were excluded from molecular analysis due to their failure to amplify the *spa* gene using the described primers or poor sequence quality for *spa* type assignment despite repeated sequencing. BURP analysis grouped 19 *spa* types into 6 *spa*-clonal complexes (*spa*-CCs) and 15 *spa* types were singletons. Among toddlers with *spa* typed isolates, 25% (19/76) were colonised with one *spa* type, while 7% (5/76) were colonised with different *spa* types on at least two of the sampled sites which were positive for *S. aureus*. One rural case toddler was colonised with *spa* type t062 on lesional skin and anterior nares, and with *spa* type t1399 on non-lesional skin which belongs to the same *spa*-CC. The most frequent *spa* types were *spa*-CC002/t002 (*spa*-CC/*spa* type; 8%), *spa* cluster 4/t272 (9%), *spa* cluster 6/t174 (14%) and *spa* cluster 5/t1476 (18%). Furthermore, we identified four new (t15783, t18354, t18750 and t19774) and one unassigned *spa* types (i.e., txAC).

### Distribution of *S. aureus spa* clonal lineages across locations by AD disease and severity

**Fig. 2:**
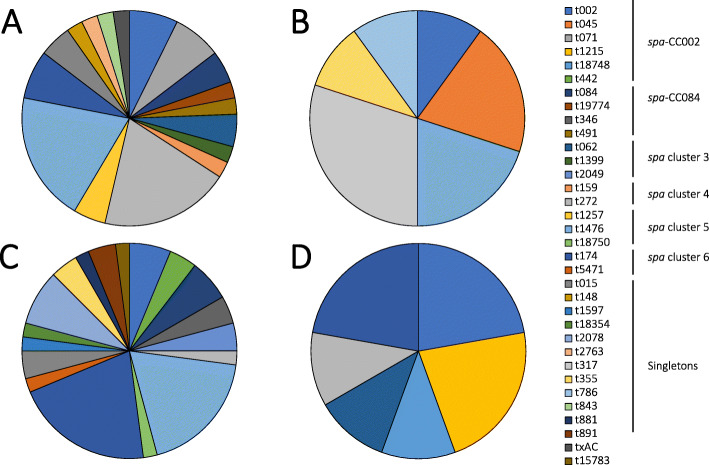
Distribution of *spa* types by disease phenotype stratified by location. **a** rural case, (**b**) rural control, (**c**) urban case, and (**d**) urban control. Percentages were calculated by the number of isolates for a *spa* type divided by the total number of *spa* types in each group

**Fig. 3:**
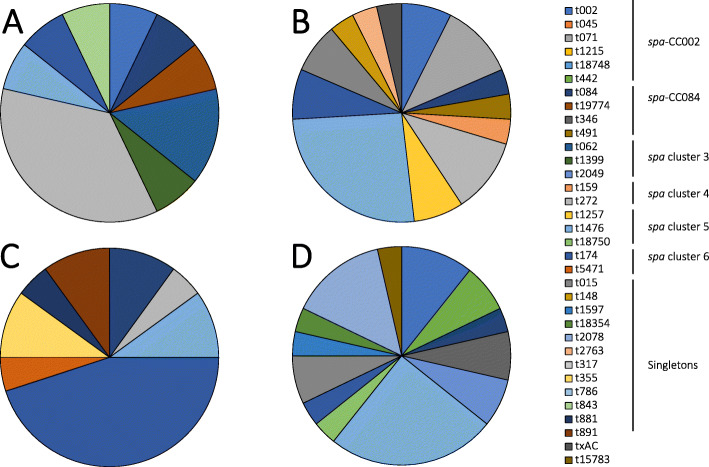
Distribution of *spa* types by disease severity. **a** rural moderate, (**b**) rural severe, (**c**) urban moderate, and (**d**) urban severe. Percentages were calculated by the number of isolates for a *spa* type divided by the total number of *spa* types in each group

The rural and urban toddlers were colonised by different *S. aureus spa* clonal lineages. The *spa* cluster 4 was frequently identified among rural toddlers (18% [9/51] vs. 4% [2/57], *p* = 0.015) and *spa* cluster 6 in urban toddlers (23% [13/57] vs. 6% [3/51], *p* = 0.013) compared to their respective counterparts based on all sampled sites (Table 4). The diversity of *spa* types among cases was higher compared to controls in both locations (Fig. 2). Moreover, comparative analysis revealed that there was an overall significant difference in the distribution of *spa* clonal lineages between urban cases and controls (*p* = 0.009), with *spa* cluster 5/t1476 and *spa* cluster 6/t174 identified as predominant among cases. There was no overall difference between rural cases and controls (*p* = 0.224), albeit, *spa* cluster 4/t272 and *spa* cluster 5/t1476 were the dominant *spa* clonal lineages among cases with no single most dominant *spa* clonal lineage among controls (Fig. 2). We also noted a significant difference in the distribution of *spa* clonal lineages among urban cases based on AD severity (*p* = 0.001). In these cases, *spa*-CC002 (t002 and t442) isolates were only identified in severe AD, *spa* cluster 6/t174 was more frequent in moderate AD, and *spa* cluster 5/t1476 in severe AD. Although no significant difference was observed between AD severity and the identified *spa* types in rural cases (*p* = 0.126), *spa* cluster 3 (t062 and t1399) isolates were only detected in moderate cases while *spa* cluster 5 (t1476 and t1257) isolates predominated in severe cases (Fig. 3).

**Table 4:** Distribution of clonal lineages of *S. aureus* isolates among Umtata and Cape Town participants

*spa*-CC	Umtata			Cape Town		
	No. of isolates (%)	No. of *spa* types (%)	*spa* types (no. of isolates)	No. of isolates (%)	No. of *spa* types (%)	*spa* types (no. of isolates)
*spa*-CC002	9	3 (14)	t002 (4); **t045 (2)**; **t071 (3)**	10	4 (19)	t002 (5); **t1215 (2)**; **t18748 (1)**; **t442 (2)**
*spa*-CC084	3	2 (10)	t084 (2); **t491 (1); t19774 (1)**	5	2 (10)	t084 (3); **t346 (2)**
*spa* cluster 3	3	2 (10)	t062 (2); **t1399 (1)**	3	2 (10)	t062 (1); **t2049 (2)**
*spa* cluster 4	9	2 (10)	**t159 (1)**; t272 (8)	2	1 (5)	t272 (2)
*spa* cluster 5	12	2 (10)	t1476 (10); **t1257 (2)**	10	2 (10)	t1476 (9); **t18750 (1)**
*spa* cluster 6	3	1 (5)	t174 (3)	13	2 (10)	t174 (12); **t5471 (1)**
Singletons	10	7 (33)	t015 (2); **t148 (1)**; **t2763 (1); t317 (3);** t355 (1); **t786 (1); t843 (1)**	13	7 (33)	t015 (2); **t18354 (1); t1597 (1); t2078 (4);** t335 (2); **t881 (1); t891 (2)**
Unaligned/*spa* types with unknown repeat succession	2	2 (10)	txAC (1)	1	1 (5)	**t15783 (1)**
Total	51	21		57	21	

Bold text indicates *spa* types that were identified in only one location

## Discussion

We conducted a cross-sectional, case-control study to determine the molecular epidemiology of *S. aureus* colonising the skin and nasal cavity of AD-affected and healthy South African AmaXhosa toddlers. We observed a higher prevalence of colonisation in cases compared to controls, regardless of geographic location. The distribution of *S. aureus spa* clonal lineages differed between rural-urban settings and differentially associated with AD disease and severity. Moreover, determinants of *S. aureus* colonisation varied across the rural-urban settings.

The pathogenesis of AD is characterised by epidermal barrier defects and activation of inflammatory responses leading to impaired clearance of skin pathogens and a decrease in skin microbial diversity [10]. *S. aureus* dominance is consistently linked with acute AD flares and severe forms of the disease [29, 30]. We noted a higher prevalence of *S. aureus* colonisation among cases compared to controls which was independent of geographic location (55% vs. 13 and 70% vs. 35% in rural and urban locations, respectively). These findings are consistent with a similar study in Italy that reported a prevalence of 57% vs. 20% in cases compared to controls [31]. Therefore, these findings support the relationship between *S. aureus* predominance and AD, regardless of population and location [9, 31].

AD-lesional skin has been shown to be more susceptible to *S. aureus* colonisation compared to AD-uninvolved, non-lesional skin, with a reported prevalence of colonisation of 23–70% vs. 6–39% [9, 32, 33]. Similarly, we noted a higher frequency of colonisation on lesional skin compared to non-lesional skin among urban and rural cases. Furthermore, similar colonisation rates on lesional skin and anterior nares have been reported in AD, with *S. aureus* nasal colonisation suggested as the main source of the increased skin colonisation in AD [9, 34]. However, we observed that lesional skin was more frequently colonised compared to the anterior nares among rural cases, suggesting a non-nasal source of *S. aureus* for the increased colonisation on lesional skin in rural AD or transient nasal colonisation [31, 35].

Skin barrier dysfunction in AD lesions, particularly in severe AD, has been correlated with increased *S. aureus* colonisation [36, 37]. In agreement with recent studies [9, 33, 37], we noted a higher prevalence of colonisation based on all sampled sites in cases with severe AD, however, this was limited to urban cases and not rural cases. Geographical location has been postulated to influence microbial colonisation and may explain the varied susceptibility of geographical populations to skin pathologies [38]. In this regard, the study communities each represent a geographic population that is uniquely affected by *S. aureus* colonisation in the pathophysiology of AD. Moreover, the rural and urban populations, regardless of disease, are generally different populations with distinct sensitisation patterns to environmental exposures [16] and inflammatory immune responses [39]. These may in turn affect microbial colonisation and the contribution thereof to disease pathogenesis and pathophysiology.

Risk factors for bacterial colonisation on the skin and nasal cavity differ with rural-urban living [40, 41]. The association between *S. aureus* colonisation and AD is well studied, with some studies reporting colonisation preceding the onset of clinically appreciable AD in toddlers and further associated with disease severity [11]. Consistent with previous reports [30], having AD in both communities was associated with *S. aureus* colonisation. Exposure to air pollutants has also been associated with increased skin barrier damage [42] which increases the propensity to *S. aureus* colonisation [43]. In rural toddlers, we observed that living in a house that uses kerosene and paraffin which release fine air particulates [44] was associated with increased *S. aureus* colonisation. However, exposure to the burning of wood/coal or outdoor fire, which also release fine air pollutants that may induce cutaneous irritation was associated with reduced *S. aureus* colonisation in rural toddlers. The effect of environmental air pollutants in children is a function of exposure time [45]. Although the toddlers are living in homes that use wood/coal or an outdoor fire for cooking and heating, they might have limited exposure to the produced particulates which restrict the possible effect on skin irritation and susceptibility to *S. aureus*. Electricity and biogas are relatively “clean fuels” with minimal air pollution emission at the household level [46]. In contrast, we found that rural living in an electrified house that also utilises gas increased the risk of *S. aureus* colonisation. Animals are reservoirs for human *S. aureus* colonisation [47], however, we found that rural toddlers living in a house with farm animals were associated with a reduced risk of *S. aureus* colonisation. Similarly, this finding could be due to the absence of direct interaction between toddlers and animals hence there are no animal-to-human *S. aureus* transmission events. Nonetheless, AD remained a risk factor while living in a house that uses wood and coal was protective against *S. aureus* colonisation in rural toddlers in the multivariate regression model. These findings highlight the importance of the immediate environment in shaping bacterial colonisation dynamics and the potential implication thereof in AD pathogenesis.

In addition to microbial colonisation, geographic location also determined the genotype of the colonising bacteria [48]. We noted heterogeneity in the distribution of the colonising *spa* clonal lineages based on geographic location, with rural toddlers mostly colonised by *spa* types belonging to the *spa* cluster 4 (previously associated with MLST CC121, Table S1) [49] while urban toddlers were predominantly colonised with *spa* cluster 6 (CC1) isolates [50]. This is similar to studies that suggest that location may play a role in the colonisation dynamics of childhood skin and nares [1, 15, 51, 52]. Furthermore, urban cases and controls exhibited distinct *S. aureus spa* clonal lineages, however, there was no difference in the distribution of *S. aureus* lineages between rural cases and controls. These findings suggest that the rural-urban locations provide a specific niche for the selection of certain *S. aureus* clonal lineages which sequentially influence the population structure in these settings, and associated colonisation dynamics. Future studies are essential to investigate site-specific features in this cohort that contribute to the observed *S. aureus* population structures and their association with disease phenotype.

The relationship between disease severity and the clonal lineages of the colonising *S. aureus* isolates is unclear with some studies reporting an association between specific clonal lineages and AD severity [15, 51] and others demonstrating none [34, 53]. In spite of this, we noted different distributions of *S. aureus* clonal lineages depending on AD severity among urban cases. Here, *spa* clonal lineages *spa* cluster 5 (CC5) [54] and *spa* cluster 6 (CC1) [50] were the most common in severe and moderate AD, respectively. The *spa*-CC002 (CC5) [15] isolates were only detected in severe AD cases. These findings are in agreement with a study in Spanish children which reported a predominance of CC5 isolates in severe AD [51] and another on the predominance of CC1 isolates in moderate AD [34], but in contrast to a report of the predominance of CC5 in moderate disease among Canadian children with AD [15]. There was a difference in the distribution of *spa* clonal lineages among rural cases based on disease severity. Albeit, *spa* cluster 3 (CC5) [49, 55] isolates were only identified in rural cases with moderate AD and *spa* cluster 6 (CC1) [50] isolates were frequent in rural cases with severe AD. The predominance of *spa* cluster 3 (CC5) isolates is similar to that noted in moderate AD elsewhere [15] while that of *spa* cluster 6 (CC1) isolates in severe AD is in contrast to previous reports of the high prevalence of CC1 isolates in children with moderate AD [34]. The contrasting predominance of *S. aureus* clonal lineages based on disease severity across the rural-urban communities emphasises the importance of the environment in the contribution of bacterial clonality in disease. Therefore, more investigations are needed to determine if certain *S. aureus* clonal lineages are associated with differential AD disease severity and the concomitant contribution to AD and disease severity.

Our data are subject to a few limitations. BURP analyses are limited to *spa* types with a cut-off ≤ 5 repeats, which excludes *spa* types with the number of repeats below the set parameter [28]. Therefore, *spa* type t15783 was excluded from BURP clustering analyses. Secondly, we predicted the corresponding MLST sequence types (STs) and CCs of the *S. aureus spa* types identified in this study by extrapolating data from previous studies (Additional file 3: Table S3). Furthermore, 14% (17/125) of the isolates were untypeable which highlights the need for whole-genome sequencing (WGS) to provide both *spa* and MLST data for detailed characterisation [1].

## Conclusion

Our study shows that toddlers with AD are more frequently colonised with *S. aureus* compared to non-AD controls. The genetic background of colonising *S. aureus* is a unique signature of AD and disease severity, however, this is largely dependent on rural-urban living. These findings highlight the importance of geographic location on the colonisation epidemiology and population structure of *S. aureus* as well as the associated colonisation determinants in childhood health and AD disease in South Africa. Future studies are planned to examine the mechanisms within the rural-urban environments that contribute to *S. aureus* colonisation dynamics and the association thereof with AD and disease severity. This information will provide insights into population-specific therapeutic strategies that may be harnessed in the restoration of microbial diversity in AD-affected toddlers.

## Supplementary Information

**Additional file 1: Table S1**. Participant colonisation among all, rural and urban cases and controls. This table is showing the distribution of *S. aureus* colonisation in AD and non-AD toddlers in the rural and urban locations.

**Additional file 2: Table S2**. Colonisation in cases stratified by disease severity among all, rural and urban cases. This table is showing the distribution of *S. aureus* colonisation based on disease severity in AD toddlers in the rural and urban locations.

**Additional file 3: Table S3**. Extrapolated MLST sequence types and clonal complexes for *spa* types identified in the present study. This table is correlating the *spa* types identified in this study to MLST clonal complexes and sequence types reported in previous studies.

## Data Availability

The datasets used and analysed during the current study are available from the corresponding author on reasonable request and ethical approval.

## Abbreviations

AD: Atopic dermatitis
BURP: Based Upon Repeat Pattern
CC: Clonal complex
CI: Confidence interval
MLST: Multi-locus sequence typing
*nuc*: Thermonuclease
OR: Odds ratio
aOR: Adjusted odds ratio
STGG: Skim milk-tryptone-glucose-glycerol
SD: Standard deviation
SAFFA: South African Food Allergy study
SCORAD: Scoring of atopic dermatitis
Spa: Staphylococcus protein A
Spa-CC: Spa clonal cluster
ST: Sequence type
