# Supplementary material for: Molecular epidemiology of Staphylococcus aureus in African children from rural and urban communities with atopic dermatitis
Source: BMC Infect Dis. 2021 Apr 13;21:348. doi: 10.1186/s12879-021-06044-4 (PMC8045247; doi:10.1186/s12879-021-06044-4)
Supplement: Supplementary file 1 — Additional file 1: Table S1. Participant colonisation among all, rural and urban cases and controls. This table is showing the distribution of S. aureus colonisation in AD and non-AD toddlers in the rural and urban locations. [file 12879_2021_6044_MOESM1_ESM.docx]

**Additional file 1: *S. aureus* colonisation based on AD and health.**

**Table S1. Participant colonisation among all, rural and urban cases and controls.**

|  | Total | | | Umtata | | | Cape Town | | |
| --- | --- | --- | --- | --- | --- | --- | --- | --- | --- |
|  | Case, *n* (%) | Control, *n* (%) | p-value | Case, *n* (%) | Control, *n* (%) | p-value | Case, *n* (%) | Control, *n* (%) | p-value |
| Lesional skin | 42 (42) |  |  | 22 (39) |  |  | 20 (48) |  |  |
| Non-lesional skin | 32 (33) | 10 (12) | 0.001 | 16 (29) | 5 (10) | 0.011 | 16 (38) | 5 (16) | 0.04 |
| Anterior nares | 28 (28) | 12 (15) | 0.028 | 9 (16) | 5 (10) | 0.318 | 19 (44) | 7 (23) | 0.067 |
