# Supplementary material for: Molecular epidemiology of Staphylococcus aureus in African children from rural and urban communities with atopic dermatitis
Source: BMC Infect Dis. 2021 Apr 13;21:348. doi: 10.1186/s12879-021-06044-4 (PMC8045247; doi:10.1186/s12879-021-06044-4)
Supplement: Supplementary file 2 — Additional file 2: Table S2. Colonisation in cases stratified by disease severity among all, rural and urban cases. This table is showing the distribution of S. aureus colonisation based on disease severity in AD toddlers in the rural and urban locations. [file 12879_2021_6044_MOESM2_ESM.docx]

**Additional file 2: *S. aureus* colonisation based on disease severity.**

**Table S2. Colonisation in cases stratified by disease severity among all, rural and urban cases.**

|  | Total | | | Umtata | | | Cape Town | | |
| --- | --- | --- | --- | --- | --- | --- | --- | --- | --- |
|  | Moderate, *n* (%) | Severe, *n* (%) | p-value | Moderate, *n* (%) | Severe, *n* (%) | p-value | Moderate, *n* (%) | Severe, *n* (%) | p-value |
| Lesional skin | 13 (32) | 29 (51) | 0.059 | 5 (24) | 17 (49) | 0.066 | 8 (40) | 12 (55) | 0.346 |
| Non-lesional skin | 13 (30) | 19 (36) | 0.562 | 6 (27) | 10 (31) | 0.753 | 7 (33) | 9 (43) | 0.525 |
| Anterior nares | 11 (26) | 17 (31) | 0.562 | 3 (14) | 6 (18) | 0.655 | 8 (38) | 11(50) | 0.432 |
