# Supplementary material for: Molecular epidemiology of Staphylococcus aureus in African children from rural and urban communities with atopic dermatitis
Source: BMC Infect Dis. 2021 Apr 13;21:348. doi: 10.1186/s12879-021-06044-4 (PMC8045247; doi:10.1186/s12879-021-06044-4)
Supplement: Supplementary file 3 — Additional file 3: Table S3. Extrapolated MLST sequence types and clonal complexes for spa types identified in the present study. This table is correlating the spa types identified in this study to MLST clonal complexes and sequence types reported in previous studies. [file 12879_2021_6044_MOESM3_ESM.docx]

**Additional file 3: Study *spa* types and MLST sequence types**

**Table S3. Extrapolated MLST sequence types and clonal complexes for *spa* types identified in the present study.**

| ***Spa* CC** | ***Spa* type** | **Ridom repeats** | **Extrapolated ST** | **Extrapolated CC** | **Reference** |
| --- | --- | --- | --- | --- | --- |
| *spa*-CC002 | t002 | 26-23-17-34-17-20-17-12-17-16 | ST5 | CC5 | ^15,56^ |
|  | t045 | 26-17-20-17-12-17-16 | ST5, ST225 | CC5 | ^15^ |
|  | t071 | 26-23-23-17-34-17-20-17-12-17-16 | ST5 | CC5 | ^51^ |
|  | t442 | 35-17-34-17-20-17-12-17-16 | ST487 | CC5 | ^15^ |
|  | t1215 | 26-23-17-34-20-17-12-17-17-16 | ST5 | CC5 | ^57,58^ |
|  | t18748 ^a^ | 26-23-23-17-34-17-20-17-34-17-20-17-12-17-16 |  |  |  |
| *spa*-CC084 | t084 | 07-23-12-34-34-12-12-23-02-12-23 | ST15, ST18 | CC15 | ^15^ |
|  | t346 | 07-23-12-34-12-12-23-02-12-23 |  | CC15 | ^15^ |
|  | t491 | 26-23-12-34-34-12-12-23-02-12-23 | ST1036 | CC15 | ^15,50^ |
|  | t19774 | 07-23-20-12-34-12-23-02-12-23 |  |  |  |
| *spa* cluster 3 | t062 | 26-23-17-12-17-16 | ST15, ST965, ST5 | CC5 | ^49,55^ |
|  | t1399 | 26-23-17-12-17 |  |  |  |
|  | t2049 | 26-23-17-17-16 |  |  |  |
| *spa* cluster 4 | t159 | 14-44-13-12-17-17-23-18-17 | ST121 | CC121 | ^49^ |
|  | t272 | 14-44-13-12-17-17-17-23-18-17 | ST121 | CC121 | ^50^ |
| *spa* cluster 5 | t1476 | 11-10-17-34-24-34-22-25 | ST8 | CC5/CC8 | ^54,59^ |
|  | t18750 | 11-10-17-34-24-22-25 |  |  |  |
|  | t1257 | 11-19-34-05-17-34-24-34-22-25 | ST612 | CC8 | ^60^ |
| *spa* cluster 6 | t174 | 14-21-16-34-33-13 | ST1 | CC1 | ^50^ |
|  | t5471 | 35-21-16-34-33-13 |  |  |  |
| Singletons | t015 | 08-16-02-16-34-13-17-34-16-34 | ST45 | CC45 | ^15,56,61^ |
|  | t317 | 08-17-23-18-23-18-17 | ST121 |  |  |
|  | t355 | 07-56-12-17-16-16-33-31-57-12 | ST152 | CC152 | ^62,63^ |
|  | t786 | 07-12-21-17-13-34-34-33-34 | ST88 | CC88 | ^61^ |
|  | t891 | 26-23-13-23-31-05-17-25-17-25-28 | ST22 | CC22 | ^64,65^ |
|  | t881 | 07-06-17-34-34-22-34 | ST1370 | CC20 | ^66^ |
|  | t843 | 04-82-17-25-17-25-25-16-17 |  | CC130 | ^67^ |
|  | t148 | 07-23-12-21-12-17-20-17-12-12-17 | ST1434 | CC72 | ^56^ |
|  | t1597 | 15-12-17-20-17-12-12-17 |  |  |  |
|  | t2078 | 04-13-21-12-17-20-17-12-17-17 |  |  |  |
|  | t2763 | 26-13-17-34-16-13 |  |  |  |
|  | t15783 | 07-23-21-24-33-17-17 |  |  |  |
| *spa* types with unknown repeat succession | txAC | r26-r08-r02-r05 |  |  |  |

MLST, multilocus sequence typing; ST, sequence type; CC, clonal cluster; ^§^Underlined text indicates sequence types that were obtained from the *spa* server (<http://spaserver.ridom.de/>).
